# Supplementary material for: MetaQTL: a package of new computational methods for the meta-analysis of QTL mapping experiments
Source: BMC Bioinformatics. 2007 Feb 8;8:49. doi: 10.1186/1471-2105-8-49 (PMC1808479; doi:10.1186/1471-2105-8-49)
Supplement: Additional File 7 — MetaQTL Package : jar file and tutorial. This Zip archive contains both the MetaQTL JAR file and the files of the tutorial. [file 1471-2105-8-49-S7.zip › org.inra.metaqtl/doc/org/metaqtl/util/class-use/OffspringManager.HaldaneWaddingtonProportion.html]

Uses of Class org.metaqtl.util.OffspringManager.HaldaneWaddingtonProportion


|  |  |  |
| --- | --- | --- |
| **Overview** \| **Package** \| **Class** \| **Use** \| **Tree** \| **Deprecated** \| **Index** \| **Help** | |  |
| PREV   NEXT | **FRAMES**    **NO FRAMES**     **All Classes** |  |


---


## **Uses of Class org.metaqtl.util.OffspringManager.HaldaneWaddingtonProportion**

| Packages that use OffspringManager.HaldaneWaddingtonProportion | |
| --- | --- |
| **org.metaqtl.util** |  |

Uses of OffspringManager.HaldaneWaddingtonProportion in org.metaqtl.util

| Methods in org.metaqtl.util with parameters of type OffspringManager.HaldaneWaddingtonProportion | |
| --- | --- |
| `static double` | `OffspringManager.selfingIndividualInformation(OffspringManager.HaldaneWaddingtonProportion p)`             This routine returns the individual means amount of information for the given generation propertions for a self-fertilization design. |
| `static void` | `OffspringManager.selfingRecurrence(OffspringManager.HaldaneWaddingtonProportion p, double r)`             Given the current proportion C,D,E,F and G and the recombination rate r this routine computes the next generation proportions following the recurrence formula of Haldane and Waddington, 1931, Inbreeding and linkage, Genetics 16:357-374 (see section self-fertilization p358). |

---


|  |  |  |
| --- | --- | --- |
| **Overview** \| **Package** \| **Class** \| **Use** \| **Tree** \| **Deprecated** \| **Index** \| **Help** | |  |
| PREV   NEXT | **FRAMES**    **NO FRAMES**     **All Classes** |  |


---
